# Relationship of Red Blood Cell Distribution Width with Cancer Mortality in Hospital

**DOI:** 10.1155/2018/8914617

**Published:** 2018-11-14

**Authors:** Jinmeng Li, Xiaoning Yang, Junfeng Ma, Fanghua Gong, Qiongzhen Chen

**Affiliations:** ^1^School of Pharmacy, Wenzhou Medical University, Wenzhou 325000, China; ^2^College of Life and Environmental Science, Wenzhou University, Wenzhou 325000, China

## Abstract

**Background:**

Red blood cell distribution width (RDW) is a clinical index used to make early diagnosis and to monitor treatment effects in iron deficiency anemia. Recently, several studies have suggested that RDW was associated with mortality from various cancers; however, there has been little evidence regarding RDW and cancer as a whole. Therefore, the purpose of our study was to investigate the relationship of RDW and overall cancer mortality in hospital.

**Methods:**

We extracted patient data from the Multiparameter Intelligent Monitoring in Intensive Care Database III version 1.3 (MIMICIII.1.3). RDW was measured prior to hospital admission. Patients older than 18 who were diagnosed with malignant tumors were included. The primary outcome was cancer mortality in hospital. Logistic regression and multivariate analysis were used to assess the association between the RDW and hospital mortality.

**Result:**

A total of 3384 eligible patients were enrolled. A positive correlation was observed between RDW and overall cancer mortality. Patients with higher RDW (14.4-16.3%, 16.4-30.5%) were at greater risk of death than the patients with RDW in the reference range (11.5-14.3%). On multivariate analysis, when adjusted for age and gender, the adjusted OR (95% CIs) in the mid-RDW group and high-RDW group were 1.61 (1.28, 2.03) and 2.52 (2.03, 3.13), respectively, with the low-RDW group set as the baseline. Similar trends were also observed in the model adjusted for other clinical characteristics. This suggested that elevated RDW was related to increased risk of cancer mortality, and RDW may play an important role in the prediction of short-term mortality after hospitalization in cancer patients.

**Conclusion:**

Elevated RDW was associated with overall cancer mortality. To a certain extent, RDW may predict the risk of mortality in patients with cancers; it was an independent prognostic indicator of short-term mortality after hospitalization in cancer patients.

## 1. Introduction

Cancer imposes a serious disease burden worldwide, with high incidence and mortality [1]. The international agency for research on cancer affirmed that, as the world's population ages, the number of cancer deaths worldwide will continue to increase [2]. The top 10 tumors were cancers of the lung, esophagus, liver, cervix, stomach, breast, colon-rectum, lymphocytes, nasopharynx, and ovary. Five-year survival rates for all-combined cancer were only 30.82% [3]. The primary methods of cancer treatment are surgical treatment, chemotherapy, and radiotherapy; however, even with all these advances, a large number of patients still have poor prognosis [4–6].

Considering the high incidence of cancer and its poor prognosis, it would be of great significance to find effective clinical predictors of mortality in cancer. Recently, several studies have reported that red blood cell distribution width (RDW) was associated with mortality in various cancers; however, there was substantially less evidence regarding RDW and all-combined cancer [7–10]. Many factors that could affect long-term prognosis of cancers have been identified, but there are relatively few identified factors affecting short-term prognosis.

The red blood cell distribution width (RDW) is a parameter that reflects the degree of heterogeneity of erythrocyte volume; it is traditionally used in hematology laboratories to help classify the anemia [7]. Nonetheless, recent evidence has shown that RDW was associated with human diseases, including cardiovascular diseases [8, 9], venous thromboses [10], liver diseases, and kidney failures [11, 12], as well as with various cancers [13]. Several studies have reported that RDW predicted the mortality of various cancers, including cancers of the lung [14, 15], stomach, colon, and endometrium [16–18]. Thus, there is a close relationship between RDW and cancer mortality. However, evidence of the role of RDW in all-combined cancer remains scarce, and the short-term prognostic value of RDW in terms of mortality remains unclear. Therefore, studying the relationship between RDW and cancer mortality is of great significance for both clinical diagnosis and patient short-term prognosis.

Therefore, we designed this study to evaluate the relationship between RDW and cancer mortality by extracting and analyzing data from the database of MIMIC-III V1.3 and predicting the short-term prognostic value of RDW in all-combined cancer mortality.

## 2. Methods

### 2.1. Data Source

Our study was based on the Multiparameter Intelligent Monitoring in Intensive Care Database III version 1.3 (MIMIC-III V1.3), a free public resource. The database includes more than 40,000 pieces of deidentified and health-related data, associated with admissions to Beth Israel Deaconess Medical Center (Boston, MA, USA) between 2001 and 2012 [19]. The database was established by the Massachusetts Institute of Technology (MIT, Cambridge, MA, USA) and Beth Israel Deaconess Medical Center. To protect privacy, all patients were deidentified.

### 2.2. Population Selection Criteria

A total of 3384 admissions were recorded. Eligible people met the following criteria: older than 18 years of age; malignant tumor confirmed by ICD-9 disease coding; and time of hospitalization > 2 days. Patients were excluded if >5% of their individual data were missing or if hospital biopsy revealed hematological malignancy.

### 2.3. Data Extraction

We extracted patient data from MIMIC-III V1.3 using Structured Query Language (SQL) with PostgreSQL (version 9.6). The data extracted were patient identifiers, demographic parameters, clinical parameters, and laboratory parameters. Patient identifiers and demographic parameters included age, gender, and ethnicity. We extracted the following clinical parameters: systolic blood pressure (SBP); diastolic blood pressure (DBP); heart rate; respiratory rate; and comorbidities including atrial fibrillation (AF); congestive heart failure (CHF); renal and liver diseases; valvular disease; and stroke and pneumonia. Laboratory parameters extracted included the following: body mass index (BMI); white blood cell count (WBC); platelet count; hematocrit; hemoglobin; blood urea nitrogen (BUN); serum anion gap; bicarbonate; creatinine; and glucose. A sequential organ failure assessment (SOFA) score was also calculated to assess the severity of illness. Hospital mortality was the primary outcome. The baseline characteristics were extracted at 24 h after hospital admission.

### 2.4. Statistical Analysis

Categorical variables were presented as percentage and variances were analyzed by the Chi-square test. Continuous variables were expressed as mean (SD) or IQR and the Kruskal-Wallis test was performed for variance comparisons. The association of RDW and cancer mortality was tested using logistic regression and results were presented as the adjusted odds ratio (OR) and associated 95% confidence interval (CI).

In order to determine whether the RDW was independently associated with cancer mortality, two multivariable analysis models were established on the basis of RDW groups. In model I, we adjusted only for age and gender. In model II, we adjusted for age and gender, as well as for SBP, DBP, BUN, hemoglobin, serum sodium, potassium, platelet, hematocrit, anion gap, renal disease, liver disease, stroke, heart rate, pneumonia, and respiratory rate.

Meanwhile, we performed subgroup analysis to determine whether the effects of RDW varied between various subgroups. Chronic obstructive pulmonary disease (COPD), acute respiratory distress syndrome (ARDS), coronary atherosclerotic heart disease (CAD), and renal replace therapy (RRT) were also included. Our statistical analyses were performed on Empowerstats version 2.17.8 and R software (version 3.42). A two tailed P value <0.05 was considered statistically significant.

## 3. Results

### 3.1. Subject Characteristics

A total of 3384 eligible cancer patients were enrolled. According to RDW value, patients were divided into three groups (low, mid, and high). A total of 1117 (33%) patients were in the low-RDW group (11.5 < RDW < 14.3), 1112 (32.8%) were in the mid-RDW group (14.4 < RDW < 16.3), and 1155 (34.1%) were in the high-RDW group (16.4 < RDW < 30.5). Selected characteristics and laboratory data in the RDW groups are displayed in Table 1.

Characteristics such as gender and body mass index (BMI) showed little difference among groups. Patients in the higher RDW group more likely have higher blood pressure, heart rate, and respiratory rate. Patients with higher RDW had more comorbidities, including atrial fibrillation (AF), congestive heart failure (CHF), valvular disease, renal and liver disease, stroke, and pneumonia. They also had higher white blood cell counts (WBC), blood urea nitrogen (BUN), serum anion gap, creatinine, and sequential organ failure assessment (SOFA) scores and were more likely to use renal replacement therapy (RRT) than those with lower RDW. However, platelet count, hematocrit, hemoglobin, serum bicarbonate, and glucose were lower in patients with higher RDW than patients in other groups.

### 3.2. Association between RDW and Cancer Mortality

**Figure 1:**
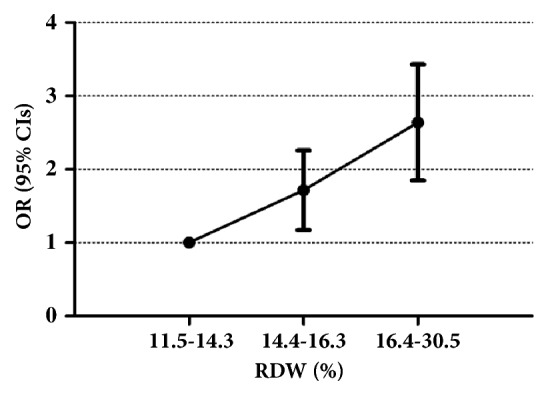
OR (95% CIs) for cancer mortality across with RDW.

We considered RDW as a continuous variable.  Figure 1 shows the association between RDW and cancer mortality. A positive correlation was observed, suggesting that patients with higher RDW were at greater risk of cancer mortality.

**Figure 2:**
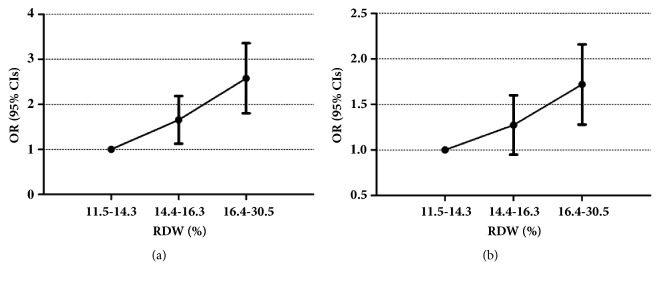
OR (95% CIs) for cancer mortality across fitted group of RDW (fitted group: model I and model II).

On multivariate analysis, when we adjusted for age and gender, the adjusted ORs (95% CIs) in the mid-RDW group and high-RDW group were 1.61 (1.28, 2.03) and 2.52 (2.03, 3.13), respectively, and the low-RDW group was set as the baseline (OR (95% CIs) = 1.0). Higher OR (95% CIs) indicated a greater risk of mortality. Meanwhile, RDW was also independently associated with cancer mortality when adjusted for age, gender, BUN, hemoglobin, sodium, potassium, platelet, hematocrit, anion gap, renal disease, liver disease, stroke, heart rate, pneumonia, SBP, DBP, and respiratory rate (Figure 2, Table 2).

### 3.3. Subgroup Analyses

The relationship between RDW and the cancer mortality was similar in most strata (Table 3). Patients in most subgroups had no differences in terms of risk of cancer mortality according to RDW. Significant differences could be observed in COPD and RRT subgroups; patients who had a high RDW only, without COPD or RRT, had a higher risk of cancer mortality, whereas if a patient had COPD or RRT, RDW had little effect on cancer mortality. We also made a subgroup analysis of the types of tumor. We selected three tumors with the highest mortality in the data we extracted from MIMICIII.1.3 database and analyzed the effect of RDW on the mortality of these three tumors by multivariate analysis. The three different types of tumors were lung cancer, gastroenteric tumor, and breast cancer. The associations between RDW and the mortality of three different types of tumors were presented in Table 4. According to the OR (95% CIs) of different groups, RDW can positively affect the mortality of three types of tumors.

## 4. Discussion

We found a positive correlation between RDW and cancer mortality, with higher RDW associated with increased risk of cancer mortality, showed RDW may be used to predict the mortality of tumor and the risk assessment of tumor patients. On multivariate analysis, the model only adjusting for age and gender suggested that higher RDW correlated with increased risk of hospital mortality. Similar trends could also be observed in the model adjusted for a greater number of characteristics, suggesting that RDW may be an effective tumor prognostic factor. Although several previous studies suggested that RDW was associated with mortality in various cancers [14–18, 20, 21], evidence to solidify the relationship remains rare. Moreover, most studies only demonstrated associations between the RDW and a single type of cancer; the relationship between RDW and all-combined cancer mortality remains unclear, and the role of RDW in tumor short-term prognosis is also very vague. Therefore, we evaluated the relationship between RDW and all-cancer mortality and proved the effect of RDW in tumor short-term prognosis.

There are many factors affecting the risk of cancer mortality. Our study demonstrated that RDW was an independent risk factor using multivariate analysis and adjusting for age and gender. This has substantial implications for clinical diagnosis and patient short-term prognosis. Given the results of our study, a positive relationship could be observed, and patients with higher RDW had an increased mortality rate. On subgroup analysis, we found the same positive correlation between RDW and cancer mortality in most strata. We infer that RDW could be a major short-term prognostic marker of hospital mortality for cancer patients. However, the explanations and mechanisms for the relationship between RDW and cancer mortality require more research to clarify.

Many studies have shown that inflammation was associated with tumor progression and metastasis [22–24]. Recently, RDW was reported as an emerging novel biomarker for systemic inflammation [25, 26]. Many other hematological parameters, including neutrophil/lymphocyte ratio (NLR) [27, 28], platelet/lymphocyte ratio (PLR) [28], lymphocyte/monocyte ratio [29], C-reactive protein [27], and interleukin-6 [30], all closely related to the inflammatory response and anemia, also have been reported to play a prognostic role in cancers. In addition, the RDW can be used as an important index for early diagnosis of iron deficiency anemia and may provide reference for clinical prevention of iron deficiency anemia [31]. Meanwhile, some studies reported that anemia was related to worse outcome in some type of cancers [31–33]. Anemia could also be caused by a systemic inflammation response in some cancers [34]. Therefore, we speculate that RDW could affect cancer mortality by influencing the inflammatory reaction and anemia. Further study should be conducted to test this hypothesis.

The primary strength of the present study is that we demonstrated a relationship between RDW and all-combined cancer mortality, with significant implications for clinical prognosis and the short-term prognosis of cancer patients. In addition, we used a free public database MIMIC-III V1.3 and extracted and analyzed sufficient patient data.

Nevertheless, there were a few limitations to this study. First, our analysis was a single-center retrospective analysis. Therefore, more prospective multicenter studies are needed. Second, RDW was measured only after admission, and a single measurement of RDW was not sufficient to reflect the degree of heterogeneity of erythrocyte volume. Third, we did not establish a predictive model for analyzing the relationship between RDW and cancer mortality, requiring further investigation. Finally, individual patient data were missing and outliers were present, possibly influencing our results.

## 5. Conclusions

We found a positive correlation between RDW and cancer mortality by extracting and analyzing a large amount of data, indicating that increased RDW was related to high-risk of mortality. RDW was an independent prognostic indicator of short-term mortality after hospitalization in cancer patients.

## Figures and Tables

**Table 1:** Baseline characteristics of the study population.

**Characteristics**	**RDW (**%**)**			**P-value**
	11.5-14.3	14.4-16.3	16.4-30.5	
RDW start	13.5 ± 0.6	15.2 ± 0.6	18.2 ± 1.6	<0.001
Clinical parameters, n (%)	1117	1112	1155	
Age, years	64.7 ± 14.9	68.2 ± 13.9	67.0 ± 14.3	<0.001
Gender, n (%)				0.533
Male	487 (43.6)	493 (44.3)	530 (45.9)	
Female	630 (56.4)	619 (55.7)	625 (54.1)	
Ethnicity, n (%)				0.015
White	812 (72.7)	838 (75.4)	840 (72.7)	
Black	72 (6.4)	76 (6.8)	109 (9.4)	
Other	233 (20.9)	198 (17.8)	206 (17.8)	
SBP, mmHg	119.3 ± 15.5	118.1 ± 16.7	115.2 ± 16.6	<0.001
DBP, mmHg	61.8 ± 9.7	60.5 ± 10.3	59.8 ± 10.5	<0.001
Heart rate, beats/minute	87.7 ± 16.1	90.1 ± 16.2	92.1 ± 17.4	<0.001
Respiratory rate, beats/minute	18.9 ± 4.3	19.7 ± 4.5	20.0 ± 4.4	<0.001
Comorbidities				
Atrial fibrillation, n (%)	273 (24.4)	324 (29.1)	342 (29.6)	0.010
Congestive heart failure, n (%)	92 (8.2)	156 (14.0)	160 (13.9)	<0.001
Renal disease, n (%)	63 (5.6)	128 (11.5)	158 (13.7)	<0.001
Liver disease, n (%)	37 (3.3)	80 (7.2)	95 (8.2)	<0.001
Valvular disease, n (%)	380 (34.0)	415 (37.3)	462 (40.0)	0.013
Stroke, n (%)	112 (10.0)	93 (8.4)	77 (6.7)	0.015
Pneumonia, n (%)	307 (27.5)	357 (32.1)	358 (31.0)	0.046
Laboratory parameters				
Body mass index, kg/m^2^	27.3 ± 5.8	27.7 ± 6.3	27.6 ± 5.7	0.524
White blood cell count, 10^ 9^ /L	12.5 ± 6.7	13.1 ± 11.0	15.1 ± 26.9	<0.001
Platelet count, 10^ 9^ /L	246.7 ± 121.5	239.6 ± 149.0	224.1 ± 171.1	0.001
BUN, mg/dl	20.9 ± 15.6	27.6 ± 22.5	31.8 ± 25.0	<0.001
Serum potassium, mmol/L	4.2 ± 0.5	4.2 ± 0.6	4.2 ± 0.6	0.028
Hemoglobin, g/dl	11.4 ± 1.8	10.5 ± 1.7	9.8 ± 1.6	<0.001
Hematocrit, %	33.6 ± 5.3	31.2 ± 5.1	29.6 ± 4.9	<0.001
Serum anion gap, mmol/L	13.9 ± 3.0	14.3 ± 3.8	14.9 ± 3.9	<0.001
Serum bicarbonate, mmol/L	23.9 ± 3.9	23.5 ± 4.5	22.9 ± 4.6	<0.001
Serum creatinine, mg/dl	1.1 ± 0.8	1.4 ± 1.6	1.4 ± 1.3	<0.001
Serum glucose, mg/dl	148.3 ± 52.9	144.6 ± 47.4	141.0 ± 55.4	0.004
Scoring systems				
SOFA	3.8 ± 2.8	4.7 ± 3.1	5.5 ± 3.5	<0.001
Hospital expire	145 (13.0)	222 (20.0)	321 (27.8)	<0.001
Renal replace therapy	34 (3.0)	49 (4.4)	80 (6.9)	<0.001

BUN: blood urea nitrogen, SBP: systolic blood pressure, DBP: diastolic blood pressure, and SOFA: sequential organ failure assessment. Normally distributed data are presented as the mean (SD) (analysis of variance); nonnormally distributed data are presented as median (IQR) (nonparametric Wilcoxon test); and categorical variables are presented as n (%) (Chi-square test).

**Table 2:** OR (95% Cls) for all-cause mortality across fitted groups of RDW (fitted groups: model 1 and model 2).

**Exposure**	**Non-adjusted**	**Adjust I**	**Adjust II**
Clinical parameters, n	3384	3384	3346
RDW start group			
11.5 - 14.3	1.0	1.0	1.0
14.4- 16.3	1.67 (1.33, 2.10) <0.0001	1.61 (1.28, 2.03) <0.0001	1.23 (0.95, 1.60) 0.1153
16.4- 30.5	2.58 (2.08, 3.20) <0.0001	2.52 (2.03, 3.13) <0.0001	1.66 (1.28, 2.16) 0.0002
RDW start group trend	1.24 (1.18, 1.31) <0.0001	1.24 (1.18, 1.30) <0.0001	1.13 (1.06, 1.20) 0.0001

Table data: *β* (95%CI) P value / OR (95%CI) P value.

Outcome: hospital mortality.

Exposure: RDW group; RDW group trend.

Nonadjusted model adjust for none.

Adjust I model adjust for age; gender.

Adjust II model adjust for age; gender; heart rate; respiratory rate; liver disease; CAD; stroke; pneumonia; valvular disease; serum sodium; serum potassium; platelet count; crematory; anion gap; serum bicarbonate; SOFA; SIRS; renal replace therapy; scoring system.

**Table 3:** Subgroup analysis of the associations between cancers mortality and the RDW.

**RDW**	**N**	**OR (95% Cls)**	**P value**
Age, years			
19.3 - 61.0	1143	1.24 (1.17, 1.30)	<0.0001
61.0 - 74.3	1143	1.15 (1.09, 1.22)	<0.0001
74.3 - 91.4	1144	1.08 (1.02, 1.14)	0.0066
Sex, n (%)			
Male	1530	1.16 (1.11, 1.21)	<0.0001
Female	1900	1.15 (1.10, 1.20)	<0.0001
Ethnicity, n (%)			
White	261	1.20 (1.08, 1.34)	0.0007
Black	2520	1.16 (1.11, 1.20)	<0.0001
Other	649	1.15 (1.08, 1.24)	0.0001
SBP, mmHg			
71.5 - 108.3	1139	1.16 (1.11, 1.22)	<0.0001
108.3 - 122.9	1140	1.16 (1.09, 1.23)	<0.0001
122.9 - 176.6	1140	1.10 (1.04, 1.17)	0.0013
DBP, mmHg			
27.9 - 55.8	1139	1.18 (1.12, 1.25)	<0.0001
55.8 - 64.4	1138	1.12 (1.06, 1.18)	<0.0001
64.4 - 103.8	1141	1.15 (1.09, 1.22)	<0.0001
Hematocrit, %			
18.1 - 31.1	1120	1.09 (1.04, 1.14)	0.0007
31.2 - 35.9	1130	1.20 (1.13, 1.27)	<0.0001
36 - 66.7	1179	1.24 (1.16, 1.33)	<0.0001
Hemoglobin, g/dl			
6.1 - 10.3	1126	1.08 (1.03, 1.14)	0.0012
10.4 - 12	1141	1.19 (1.13, 1.27)	<0.0001
12.1 - 21.5	1162	1.22 (1.13, 1.31)	<0.0001
Respiratory rate, beats/minute			
9.9 - 17.2	1139	1.22 (1.14, 1.30)	<0.0001
17.2 - 20.8	1138	1.12 (1.06, 1.19)	0.0001
20.8 - 42.2	1139	1.12 (1.07, 1.18)	<0.0001
Serum bicarbonate, mmol/L			
6 - 22	945	1.13 (1.07, 1.19)	<0.0001
23 - 25	1078	1.23 (1.15, 1.31)	<0.0001
26 - 46	1403	1.11 (1.05, 1.16)	0.0001
Congestive heart failure			
0	3018	1.16 (1.13, 1.20)	<0.0001
1	412	1.11 (1.01, 1.22)	0.0362
Atrial fibrillation			
0	2478	1.17 (1.13, 1.22)	<0.0001
1	952	1.11 (1.05, 1.18)	0.0004
COPD			
0	3337	1.16 (1.13, 1.20)	<0.0001
1	93	0.99 (0.78, 1.24)	0.9016
Respiratory failure			
0	1995	1.16 (1.10, 1.22)	<0.0001
1	1435	1.17 (1.12, 1.22)	<0.0001
ARDS			
0	3351	1.15 (1.12, 1.19)	<0.0001
1	79	1.30 (1.07, 1.56)	0.0069
Pneumonia			
0	2392	1.18 (1.13, 1.23)	<0.0001
1	1038	1.13 (1.07, 1.19)	<0.0001
Valvular disease			
0	2160	1.13 (1.08, 1.18)	<0.0001
1	1270	1.20 (1.14, 1.26)	<0.0001
CAD			
0	2907	1.16 (1.12, 1.20)	<0.0001
1	523	1.13 (1.03, 1.23)	0.0102
Stroke			
0	3145	1.16 (1.12, 1.20)	<0.0001
1	285	1.15 (1.03, 1.29)	0.0107
Renal disease			
0	3075	1.16 (1.12, 1.20)	<0.0001
1	355	1.13 (1.02, 1.24)	0.0192
Liver disease			
0	3213	1.16 (1.12, 1.20)	<0.0001
1	217	1.13 (1.01, 1.26)	0.0343
Renal replace therapy			
0	3259	1.16 (1.12, 1.20)	<0.0001
1	171	1.06 (0.96, 1.18)	0.2569

SBP: systolic blood pressure, DBP: diastolic blood pressure, COPD: chronic obstructive pulmonary disease, ARDS: acute respiratory distress syndrome, and CAD: coronary atherosclerotic heart disease.

ORs (95% CIs) were derived from logistic regression analysis models.

**Table 4:** Subgroup analysis of the associations between the mortality of three different types of tumors and the RDW.

**RDW start group**	**Lung cancer**	**Gastroenteric tumor**	**Breast cancer**
11.5 - 14.3	1.0	1.0	1.0
14.4- 16.3	1.19 (0.94, 1.53)	1.51 (1.26, 1.90)	1.28 (1.12, 1.79)
16.4- 30.5	1.89 (1.36, 2.29)	2.01 (1.89,2.31)	1.56 (1.41,1.91)

ORs (95% CIs) were derived from logistic regression analysis models.

## Data Availability

The data used to support the findings of this study are available from MIMIC database; getting these data it requires a permission from the MIMIC database.
